# Disseminated Sclerosis

**Published:** 1917-04-07

**Authors:** 


### DISSEMINATED SCLEROSIS.

### A Knotty Problem of Causation and Treatment.

The disease—or perhaps one should write syndrome—usually known as disseminated or insular cerebro-spinal sclerosis is, though fortunately not common, at least by no means a rarity; that is to say, no reasonably busy practitioner could very well pass through his professional career without coming across one or more cases of it; and specialists in nervous diseases see it quite frequently. The symptoms have long ago been accurately catalogued, the diagnosis is not as a rule difficult, even in early cases, and the final ending can be predicted almost with mathematical certainty. In spite of the very careful study of many distinguished neurologists, the rest of the natural history of this disease is extraordinarily obscure. No one knows the cause or causes of it, no one can cure it, and no one can foretell with any accuracy what sort of course any given case of it will run.

In two recent papers in the *Edinburgh Medical Journal** Dr. Byrom Bramwell, the veteran Edinburgh neurologist, summarises the statistics of the two hundred cases which he has personally encountered during his career, and ends with some interesting speculations upon the causation of the malady. His figures, it may be stated at once, disclose little that has not long been commonly accepted about disseminated sclerosis. They are not on that account of the less value, for it is always well to take stock in the light of modern progress now and then of the "orthodox" views of any familiar medical problem.

### Prognosis not Quite Hopeless.

Dr. Bramwell starts off with a paper on prognosis, in the course of which he shows that, unfavourable as the prognosis is, there are yet cases in which permanent arrest and even apparent cure of the symptoms are known to take place. In his two hundred cases he has met with three cases which appear to be instances of complete recovery, and with a handful more in which the downward progress has been so slow that the patients have lived for as long as thirty years or even more. It is not given to many practitioners to see two hundred cases of disseminated sclerosis, and the general idea has consequently gained ground that the diagnosis of this disease is equivalent to sentence of death within a few years. To remind the profession, therefore, that recovery is possible, even though most improbable, and that the course of the disease is sometimes exceedingly slow, is to render a distinct service both to physicians and their patients.

The erratic manner in which disseminated sclerosis develops (or fails to develop) is well known, and is emphasised in every text-book. Dr. Bramwell's records provide many instances of these eccentricities. In many cases, as he says, the downward progress is from time to time interrupted by periods of improvement or complete remission of the symptoms. These periods of improvement and remission are very deceptive, for they may lead the physician to give an unduly optimistic prognosis; and if any special line of treatment is being exhibited to attribute the amelioration to that treatment. Everyone who has had much experience of disseminated sclerosis, says Dr. Bramwell, knows that, although the patients often improve and, in some instances, apparently for a time get quite well, it is only very rarely that the improvement is lasting.

### A Question of Ætiology.

For years he has believed that the patches of sclerosis in the nervous system, which underlie the symptoms of this disease, are due to some toxin distributed to the nervous system by the circulation. Remissions and recurrences are easily explained on this hypothesis by assuming that there are separate periods of recurring intoxication, produced in all probability within the body (*i.e.*, not introduced from without, as, for instance, by metallic poisoning, which some authorities have suggested as the cause of the disease). Complete recovery is also explained by assuming that no further auto-intoxication occurs. The difficulty created by this hypothesis is that no one can detect or even suggest what the assumed toxin can be; and it is with the object of throwing some light upon this point that Dr. Bramwell has carefully analysed his cases, though without any success other than that of a negative kind.

The shortest duration of any case in his series was seven months; the longest thirty-seven years. In 106 fatal cases the average duration has been ten years and eight months. Four-fifths of the patients were between sixteen and thirty-five years of age when their symptoms were first noticed; only in five did the disease begin (or appear to begin) after the age of fifty. It is this predilection of disseminated sclerosis for the flower of early manhood (and womanhood) which constitutes one of the most distressing features of this strange disease. In 129 out of 192 cases in which the status of the patient is recorded the patients were unmarried when the onset of the disease was first noticed. Neither heredity nor occupation is shown to have any influence on the malady in Dr. Bramwell's experience, though he notes the curious fact that the great majority of the patients were comfortably circumstanced and that only a very small proportion were really poor.

### A Suggestion for Further Research.

In common with the accepted authorities, he finds that venereal diseases have no influence as causes of the condition. The alleged or supposed causes were very numerous; so numerous and so varied that they have, one may say, no common factor. Closer investigation showed in many cases that the alleged or supposed causes had been actually preceded by some one or other of the

* January 1917, p. 16, and February 1917, p. 96.

early symptoms of the sclerosis. The patients were nearly always healthy, non-nervous, young men and women in good or fairly good circumstances; a very large proportion of them were engaged in household work or living in comfortable homes without any employment. These considerations certainly narrow down the field of speculation and inquiry into the causes of disseminated sclerosis; but they cannot be said to offer any positive guide to the seeker after knowledge about this serious and disabling condition. The only other hypothesis that has any adherents is the developmental, according to which the origin of the disease is to be sought in some defect of the neuroglial or nervous tissues which renders it more vulnerable than these tissues are in a normal individual. In the nature of things this is, and is likely to remain, a pure speculation; but it is worth noting, as Dr. Bramwell shows, that it does not exclude other etiological factors (*e.g.*, toxins) acting as occasional or additional causes. It possesses the further drawback that it is vague in expression as well as in conception: as an explanation it is merely a way of taking refuge in the inexplicable.

